# The median non-prostate cancer survival is more than 10 years for men up to age 80 years who are selected and receive curative radiation treatment for prostate cancer

**DOI:** 10.1186/1748-717X-2-17

**Published:** 2007-05-18

**Authors:** Paul A Blood, Tom Pickles

**Affiliations:** 1 Radiation Oncology, BC Cancer Agency and University of British Columbia, Victoria, BC, Canada; 2 Radiation Oncology, BC Cancer Agency and University of British Columbia, Vancouver, BC, Canada

## Abstract

Treatment guidelines recommend that curative radiation treatment of prostate cancer be offered only to men whose life expectancy is greater than 10 years. The average life expectancy of North American males is less than 10 years after age 75, yet many men older than 75 years receive curative radiation treatment for prostate cancer. This study used the provincial cancer registry in British Columbia, Canada, to determine median non-prostate cancer survival for men who were aged 75 to 82 years at start of radiation treatment. Median survival was found to be greater than 10 years in men aged up to 80 years at the start of their radiation treatment. This finding suggests that radiation oncologists are able to appropriately select elderly men with greater than average life expectancy to receive curative radiation treatment.

## Background

It is generally accepted that men with low and intermediate risk for prostate cancer should be treated with curative intent only if their life expectancy exceeds 10 years [1]. The average life expectancy of North American males is less than 10 years after age 75 [2], yet recent reports from the U.S. indicate that more than 35% of men with prostate cancer who are older than 75 are treated with radiation therapy [3]. Are these elderly men being treated inappropriately, or are radiation oncologists able to appropriately select for radiation treatment elderly men whose life expectancy is better than the average for their age?

The objectives of this study were to determine the life expectancy from non-prostate cancer death for men aged 75 and older who are treated with curative radiotherapy for prostate cancer, and to compare their life expectancy with that of the general male population.

## Methods

The study included men who started curative radiotherapy for prostate cancer between 1984 and 2004, who were age 75 to 82 at the date of starting the therapy. Data was taken from the British Columbia Cancer Registry, which records all cancer diagnoses and treatments in the province of British Columbia (BC), Canada [4,5]. Mortality was determined from death certificates recorded in the Cancer Registry. Death certificates were available up to December 31, 2004.

## Results

Between 1984 and 2004, 4,005 men aged 75 to 82 started radiation treatment for prostate cancer in BC. According to the risk criteria of the Canadian Consensus Guidelines [6], 56% of the men had high-risk prostate cancer, 33% had intermediate risk, and 11% had low risk. The median radiotherapy dose and fractionation was 66 Gy in 33 fractions (Range: 50 Gy in 16 fractions to 74 Gy in 37 fractions). One hundred and ten men were treated with brachytherapy.

**Figure 1:**
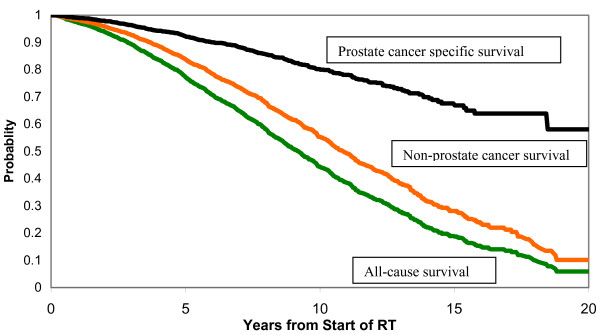
Kaplan-Meier survival functions. The top curve is prostate specific survival, the middle curve is non-prostate cancer survival and the bottom curve is all-cause survival.

Figure 1 shows the Kaplan-Meier (K-M) survival curves for deaths from prostate cancer, non-prostate cancer deaths, and deaths from all causes. Survival is measured from the start date of radiation treatment. The K-M prostate cancer survival censors deaths from non-prostate cancer and men who are still alive at the end of the study period. The K-M non-prostate cancer survival censors deaths from prostate cancer and men who are still alive at the end of the study period.

**Figure 2:**
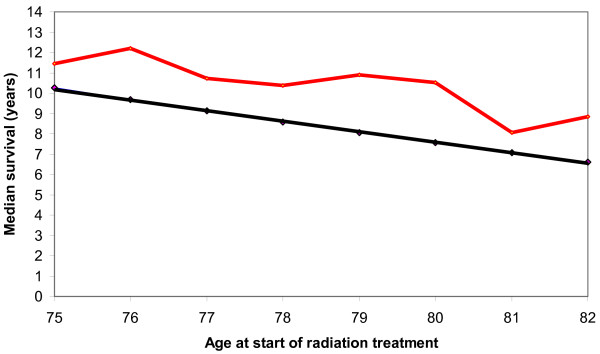
Median survival at age of starting radiation treatment. The top line is the median survival from non-prostate cancer death for men treated with radiation treatment. The bottom line is the median survival at the same age for the male population of British Columbia, Canada.

Figure 2 shows the K-M median non-prostate cancer survival by age at start of radiation treatment and the median all-cause survival for all men of the same age in the BC population. The median non-prostate cancer survival is greater than 10 years for men aged up to 80 years at start of radiation treatment. The non-prostate cancer survival of men selected for radiation treatment is consistently longer than the all-cause survival of men of the same age in the BC population.

**Figure 3:**
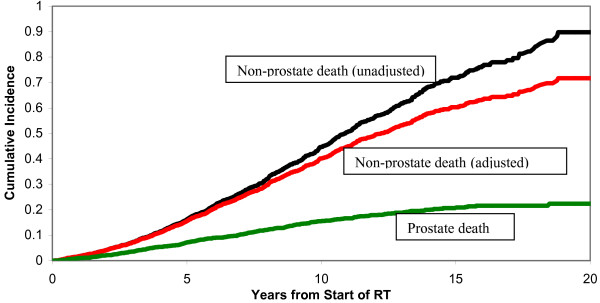
Cumulative incidence of death functions. The top line shows the cumulative incidence of non-prostate cancer death calculated using the Kaplan-Meier method without accounting for competing deaths from prostate cancer. The middle line shows the cumulative incidence after adjusting for the competing risk of death from prostate cancer. The bottom line shows the cumulative incidence of prostate cancer death.

Figure 3 shows the cumulative incidence of non-prostate cancer mortality unadjusted and adjusted for prostate cancer mortality. Deaths from prostate cancer are a competing cause of mortality with non-prostate cancer deaths. Figure 3 shows that the cumulative incidence of non-prostate cancer mortality is reduced by adjusting for prostate cancer mortality.

## Discussion

We have shown that the median survival from non-prostate-cancer deaths for men who are treated with radiation for prostate cancer is more than ten years, up to age 80 at the time of starting radiation treatment. Our results suggest that radiation oncologists are successful in selecting for curative treatment men whose life expectancy is greater than would be estimated from their age alone.

An important limitation of our analysis is that we are unable to know the life expectancy from non-prostate causes for all of the men who received radiation treatment, because of the competing cause of death from prostate cancer. However, there is no a-priori reason to believe that those men who died from prostate cancer were less healthy, and would have had a non-prostate cancer death, sooner than those men who did not die from prostate cancer. Kaplan-Meier curves cannot be adjusted for competing risks [7]. However, cumulative incidence of mortality can be adjusted for competing risks [7]. Our finding that the cumulative mortality risk is lowered after adjustment for competing risks is in accordance with the findings of Satagopan et al, who reported that the cumulative incidence of breast cancer mortality is reduced, compared to Kaplan-Meier estimates, after adjusting for death due to other causes [8].

Several studies using population health data have suggested that a significant number of elderly men diagnosed with prostate cancer are treated with radiation, versus undergoing watchful waiting or expectant management. Lu-Yao and colleagues found that Medicare beneficiaries aged 65 to 79 in Seattle had a 2.3-fold higher rate of radiation treatment during 1987 to 1990 compared to Medicare beneficiaries aged 65 to 79 in Connecticut. However, men in Seattle had the same survival from prostate cancer as men in Connecticut, despite the higher rate of radiation treatment. [9]. Using data from the Surveillance, Epidemiology and End Results (SEER) cancer registry linked to Medicare claims data, Miller et al. reported that 45% of men with low-risk prostate cancer were over-treated with radiation between 2000 and 2002, with the greatest burden of over-treatment falling on men over the age of 70 years [3].

The 2007 National Cancer Network Guidelines (NCNG) for prostate cancer state that "life expectancy estimation is critical to informed decision-making in prostate cancer, early detection and treatment". The NCNG guidelines for curative treatment are categorized according to life expectancy above and below a median survival of 10 years [10]. This 10-year rule has become accepted in medical decision-making, but while such estimation for groups is possible, it is recognized to be a challenge for individuals [11].

A decision-analytic Markov model [12] explored the life expectancy and quality of life gain (QALG) following curative radiation treatment in those aged greater than 65 years. The study concluded that "potentially curative therapy (surgery or radiotherapy) may lead to significant gains in health outcomes for men up to at least age 75 or 80 years with moderately or poorly differentiated localized prostate cancer." These gains depended on patient comorbidities.

It is clear therefore that, rather than universally applying a specific age cut-off, radiation oncologists must decide whether to recommend curative radiation treatment on a patient-by-patient basis. That decision will consider not only tumor-risk grouping based upon initial PSA test results, Gleason score and stage, but must also consider an assessment of life expectancy, as well as respecting the patient's own preferences.

Although the clinical practice upon which the current study is based did not employ a formal comorbidity scoring system, comorbidity clearly influenced the selection of patients for therapy. The impact of comorbidity on life expectancy in men with prostate cancer has been assessed: Post et al. [13] found that younger men (aged 60) with comorbidity were twice as likely to die compared to those without such comorbidity; whereas at age 74 years, comorbidity was no longer a significant factor in life expectancy. These results must be interpreted with some caution, as the mean follow-up was only 2.9 years; however, they do reinforce the importance of patient selection for curative intervention. A review of comorbidity assessment in prostate cancer [14] suggests that comorbidity assessments should be used more frequently. An electronic application for calculating a Charlson comorbidity score is available at no cost to facilitate this in daily practice [15]. Kastner and colleagues found that the Charlson comorbidity score is easy to use in everyday practice and is a significant predictor of survival for men with localised prostate cancer [16]. The present study did not employ a formal comorbidity score, but the results suggest radiation oncologists are able to appropriately judge the health and potential life expectancy of their patients.

The current study is limited by reliance on administrative data not collected for answering the study question. A potential bias may exist in the determination of cause of death: men who are followed after treatment for prostate cancer are more likely to have their death attributed to prostate cancer. In this study, this bias could lead to over-estimation of survival from non-cancer causes in men treated for prostate cancer. Penson and colleagues have assessed the accuracy of death certification for prostate cancer deaths [17]. They found that the Kappa statistic was 0.91 for agreement between the death certificate cause of death and physician assessment of the cause of death from medical records.

## Conclusion

This study suggests that radiation oncologists in British Columbia are selecting elderly patients appropriately for curative therapy, and that median non-prostate cancer survival exceeded the survival of the general population. Formal comorbidity assessments were not employed in patient assessments, but could provide additional information to guide the treatment decision-making process.

## Competing interests

The author(s) declare that they have no competing interests.

## Authors' contributions

Both authors made substantial contributions to conception and design, acquisition, analysis and interpretation of data, have been involved in drafting the manuscript, and have given final approval of the version to be published.
